# Managing Medical Emergencies in Psychiatry: An Induction for Foundation Doctors

**DOI:** 10.1192/bjo.2025.10278

**Published:** 2025-06-20

**Authors:** John Harvey, La-Dantai Henriques, Muniswamy Hemavathi, Sen Kallumpuram

**Affiliations:** 1 East London NHS Foundation Trust, London, United Kingdom; 2 Bedfordshire Hospitals NHS Foundation Trust, Luton, United Kingdom

## Abstract

**Aims:** Inpatient mental health wards present an environment in which rotating foundation doctors often have little prior exposure; with the average length of psychiatry placements in the undergraduate curriculum spanning just six and a half weeks, out of a median of 85 weeks for all clinical placements. The environment of mental health wards differs significantly from the acute hospital environment, with different staffing roles, equipment, and escalation procedures. This project aimed to assess the knowledge and confidence of existing foundation doctors in managing medical emergencies on the ward and develop an induction teaching programme for new trainees to further equip them in managing these presentations. Topics including the nature and challenges of the clinical environment, familiarisation of the emergency kit, patient population-specific conditions and escalation procedures were included in the session.

**Methods:** Teaching focussed on each of the highlighted topics was delivered to new foundation trainees during their induction. This included two facilitated desktop simulation scenarios around the recognition, investigation and initial management of opioid overdose and neuroleptic malignant syndrome and subsequent debrief and facilitated reflection. Pre- and post- questionnaires were completed using 5-point Likert scales (0–strongly disagree – 5–strongly agree) allowing participants to self-assess their knowledge and confidence across a range of domains including familiarity with emergency kit, limitations of providing emergency medical care in mental health wards, and awareness of escalation procedures and referral documentation.

**Results:** This session was run with a total of 11 participants, as part of their induction programme to the trust. Improvement in Likert score was seen across all domains with an average improvement from 1.57/5 to 4.66/5. Analysis of written feedback for the session demonstrated that participants found the interactive case scenarios to be interactive and engaging, with all participants finding the session useful and appropriate to their level of clinical training.

**Conclusion:** Feedback from this session indicated that it is well received by all participants and demonstrated improvement in self-perceived knowledge and confidence across all domains assessed. Using a blend of traditional direct teaching alongside facilitated discussions and interactive scenarios was effective in facilitating engagement across the session as demonstrated in session feedback and through the use of live audience polling during the session. This session will continue to be featured as part of the induction of new foundation doctors to support them in their preparation for caring for patients in mental health wards.

